# Discordance between self‐perceived and actual risk of HIV infection among men who have sex with men and transgender women in Thailand: a cross‐sectional assessment

**DOI:** 10.1002/jia2.25430

**Published:** 2019-12-19

**Authors:** Pich Seekaew, Supabhorn Pengnonyang, Jureeporn Jantarapakde, Ratchadaporn Meksena, Thanthip Sungsing, Sita Lujintanon, Pravit Mingkwanrungruangkit, Waraporn Sirisakyot, Sumitr Tongmuang, Phubet Panpet, Saman Sumalu, Phonpiphat Potasin, Supapun Kantasaw, Pongpeera Patpeerapong, Stephen Mills, Matthew Avery, Sutinee Chareonying, Praphan Phanuphak, Ravipa Vannaki, Nittaya Phanuphak

**Affiliations:** ^1^ PREVENTION Thai Red Cross AIDS Research Centre Bangkok Thailand; ^2^ Department of Epidemiology Mailman School of Public Health Columbia University New York NY USA; ^3^ Rainbow Sky Association of Thailand Bangkok Thailand; ^4^ The Service Workers in Group Foundation Bangkok Thailand; ^5^ Sisters Foundation Chonburi Thailand; ^6^ Caremat Organisation Chiang Mai Thailand; ^7^ Mplus Foundation Chiang Mai Thailand; ^8^ FHI 360 and USAID LINKAGES Project Bangkok Thailand; ^9^ Office of Public Health U.S. Agency for International Development Regional Development Mission Asia Bangkok Thailand

**Keywords:** men who have sex with men, transgender women, HIV prevention, risk perception, risk discordance, HIV infection

## Abstract

**Introduction:**

Low uptake of HIV testing and services, including pre‐exposure prophylaxis (PrEP), in Thai men who have sex with men (MSM) and transgender women (TGW) may be due to the inaccuracy in self‐risk assessment. This study investigated the discordance between self‐perceived HIV risk and actual risk.

**Methods:**

Data were obtained between May 2015 and October 2016 from MSM and TGW enrolled in key population‐led Test and Treat study in six community health centres in Thailand. Eligible participants were at least 18 years old, Thai national, had sex with men, had unprotected sex with a man in the past six months or had at least three male sex partners in the past six months, and were not known to be HIV positive. Baseline demographic behavioural characteristics questionnaires, including self‐perceived HIV risk, were self‐administered. Participants received HIV/STI (syphilis/gonorrhoea/chlamydia) testing at baseline. Participants who self‐perceived to have low risk, but engaged in HIV‐susceptible practices were categorized as having risk discordance (RD). Regression was conducted to assess factors associated with RD among MSM and TGW separately.

**Results:**

Of the 882 MSM and 406 TGW participants who perceived themselves as having low HIV risk, over 80% reported at least one of the following: tested HIV positive, engaged in condomless sex, tested positive for a sexually transmitted infection sexually transmitted infection (STI; or used amphetamine‐type stimulants. Logistic regression found that living with a male partner (*p* = 0.005), having never tested for HIV (*p* = 0.045), and living in Bangkok (*p* = 0.01) and Chiang Mai (*p* < 0.001) were associated with increased risk discordance among MSM. Living with a male partner (*p* = 0.002), being less than 17 years old at sexual debut (*p* = 0.001), and having a low knowledge score about HIV transmission (*p* < 0.001) were associated with increased risk discordance among TGW. However, for TGW, being a sex worker decreased the chance of risk discordance (*p* = 0.034).

**Conclusions:**

Future HIV prevention messages need to fill in the gap between self‐perceived risk and actual risk in order to help HIV‐vulnerable populations understand their risk better and proactively seek HIV prevention services.

## 1 Introduction

Thailand has made remarkable progress in reducing new HIV infections over the past years 1, and its trajectory is well‐set to achieve UNAIDS' 90‐90‐90 goals 2. Of all new HIV infections, men who have sex with men (MSM), transgender women (TGW) and male sex workers comprise more than half of the country's annual HIV incidence 1, making them a priority in HIV prevention efforts 3. In 2018, Thailand's 94‐75‐73 achievement for UNAIDS' three 90s targets 1 represented all populations in the country. However, when stratified to reflect only key populations (KPs), only 42.9% and 41.6% of MSM and TGW respectively were aware of their HIV positive status 1. These figures reflect the discrepancy in progress between the general population and KPs, especially for the first 90 target, which may suggest a low uptake of HIV testing among these individuals.

The low HIV testing uptake among MSM and TGW may stem from several problems: Thai population has a stigmatizing attitude towards people living with HIV 4, which may generate fear of HIV stigma and being labelled as HIV positive, thereby preventing people from seeking out their HIV status 5, 6, 7; systematic discrimination and stigma based on gender and sexual orientation, for instance in healthcare setting, can also deter some MSM and TGW from visiting healthcare provider and getting HIV testing 6, 7. Despite the efforts to make HIV testing, treatment, and prevention services more approachable to MSM and TGW using an active case‐finding Reach‐Recruit‐Test‐Treat‐Retain approach by KP‐led community‐based organizations 8, low uptake of HIV testing among MSM and TGW persists and may be attributed to the false premise that they are not at risk of HIV acquisition 7. Several studies have reported that the major reason provided by those who refused to take an HIV test was that they did not perceive themselves to be at risk 9, 10, 11, 12, 13, 14. However, self‐perceived risk and actual risk are not always congruent – individuals who perceive themselves as having no risk may actually participate in HIV‐risk behaviours 15, 16. Past studies suggested that health information increased the likelihood that a person would comply with health‐seeking behaviours 17, 18. This underscores the importance of sexual health knowledge and, subsequently, accurate self‐risk assessment among those who are at heightened risk of HIV. Furthermore, discrepancies between self‐perceived risk and actual risk can result in less‐than‐optimal coverage of HIV services and allocative inefficiency.

Past studies related to this incongruence were largely conducted in Western countries, thus little is known about the connection between self‐perceived risk and actual risk of HIV infection in Thailand. This manuscript describes incongruence between self‐perceived and actual risk among Thai MSM and TGW, and reports on factors associated with risk discordance among MSM and TGW in KP‐led Test and Treat study cohorts.

## 2 Methods

### 2.1 Study design and participants

This study is a sub‐study of KP‐led Test and Treat study. In 2015, we initiated a prospective observational cohort study for the KP‐led Test and Treat study with six community‐based clinic sites throughout Thailand: RSAT Bangkok, SWING Bangkok, SWING Pattaya, Sisters (Pattaya), Caremat (Chiang Mai) and RSAT Songkhla 8. The primary objectives of the study were to determine the proportion of first‐time HIV testers and median CD4 count at HIV diagnosis among MSM and TGW receiving KP‐led Test and Treat services, as well as to determine the uptake of early CD4 count testing through point‐of‐care CD4 and ART initiation through KP‐led support of ART linkages. Potential participants were recruited in the study areas through enhanced KP‐led health service outreach activities, which included incentivized case‐finding (a peer‐to‐peer communication and support system), promotion through social media channels frequented by MSM and TGW, work with the MSM and TGW “community influencers,” information tables in areas where large number of MSM and TGW frequent, and peer referral. Clients who accessed HIV counselling and testing services at each community‐based site were recruited by clinic counsellors and study staff. Furthermore, informative pamphlets about the study were distributed by community outreach workers in local areas. We recruited eligible MSM and TGW who were ages 18 years and older, were Thai nationals, had sex with men, had condomless anal intercourse with a man in the previous six months or had at least three male sex partners in the past six months, were not known to be HIV positive, and signed informed consent. Eligible MSM and TGW were enrolled from May 2015 to October 2016 and were followed for 24 months. As this is a prospective observational cohort study in which HIV cascade procedures were provided as part of the community‐based clinics' routine services, we did not limit or calculate sample size. This was done to reflect the real‐world setting, as we hope that the findings could be generalized to other high HIV burden areas.

The study was approved by the Chulalongkorn University Institutional Review Board (IRB:181/57), the Ethics Committee for Research in Human Subjects Department of Diseases Control (IRB:9/57‐678), Queen Savang Vadhana Memorial Hospital (IRB:21/2557), the Research Ethics Committee of Hatyai Hospital (IRB: 53/2560), Chonburi Provincial Public Health Office (IRB: cb0032.003/658), and Chiang Mai Provincial Public Health Office (cm0032.003.1/6609); NCT Number: NCT02383602.

### 2.2 Procedures

#### 2.2.1 Physiological assessment

Pre‐test and post‐test counselling, including risk‐reduction counselling, were provided to participants according to the standard practice at each site during participants' enrolment visit. All participants received an HIV test at baseline. Those who tested HIV negative were asked to come for HIV re‐testing every six months or sooner if they felt exposed to risk, and participants newly diagnosed with HIV were offered immediate ART regardless of CD4 count. Participants also were screened for STIs by using a nucleic acid amplification test to test for gonorrhoea and chlamydia with pharyngeal swab, urine, rectal swab and neovaginal swab (only for TGW who had undergone gender reassignment surgery). The treponema pallidum haemagglutination assay, with confirmation by venereal disease research laboratory or rapid plasma reagin test, was used to diagnose syphilis. Those who tested positive for STIs were treated. Additionally, support for partner notification was also provided at each site.

#### 2.2.2 Questionnaires

Self‐administered questionnaires were given to participants at their baseline visit to capture information on demographic profiles, behavioural risks, and HIV knowledge prior to HIV testing. Participants were asked to rate their own HIV risk as “No,” “Mild,” “Moderate” or “High” prior to receiving the counselling to assess their self‐perceived HIV risk. In addition to the baseline visit, questionnaires pertaining to behavioural risk were given at every follow‐up visit.

### 2.3 Statistical analysis

Survey about self‐perceived risk were categorized into *no*, *mild*, *moderate* and *high* risk based on the self‐reported responses. To increase the overall fitness of the analysis model, we combined *no* and *mild* groups, and recategorized people who gave these responses as perceiving themselves to be at “low” risk of getting HIV. Similarly, we combined *moderate* and *high* groups, and recategorized people who provided these responses as perceiving themselves to be at “high” risk of getting HIV. To measure the congruence or incongruence between self‐perceived and actual risk of HIV infection, participants with at least one of the following characteristics were defined as having actual risk: tested HIV positive at baseline, engaged in condomless sex in the past six months, reported to have any symptoms or were diagnosed with an STI at baseline, used amphetamine‐type stimulants (ATS) (injectable or non‐injectable), used illicit intravenous drugs in the previous six months and/or shared needles with others. Participants who reported none of these characteristics were defined as having no actual risk. In this study, we only included participants with self‐perceived “low” risk of getting HIV in the analysis. Based on previous literature, these individuals may be at higher risk of acquiring HIV 15, 16, and could require a different approach to facilitate their health‐seeking behaviour when compared to those who perceived themselves to have high risk.

The demographic characteristics of the participants, together with their baseline behaviour risk information and STI and HIV clinical characteristics, were reported overall and by gender‐specific groups (MSM and TGW) as frequency and proportion for categorical variables; mean, standard deviation (SD), median and interquartile range (IQR) for continuous variables. Comparison of continuous variables between groups was made by using a two‐sample t test or Mann‐Whitney U two‐statistic. chi‐square or Fisher's exact was used for comparison of proportion of characteristics between those whose self‐perceived risk was congruous and incongruous with their actual risk.

HIV prevalence was assessed at baseline and 95% confidence interval (95% CI) around the prevalence rate, which was calculated according to a binomial distribution. The difference in HIV prevalence between those whose self‐perceived risk was congruous and incongruous with their actual risk was tested by chi‐square.

Gender‐stratified logistic regression was performed to explore correlations between self‐perceived and actual risk of HIV infection. Assumptions about linearity of continuous covariates such as age, age at first sex, and number of sexual partners were checked by breaking the variable into quartiles and examining the odds ratio and 95% CI for each quartile. When these assumptions were not met, categorical groupings were used, and adjacent quartiles were collapsed together, if appropriate. Baseline covariates with *p* < 0.20 were included and adjusted for in multivariable models by enter method.

Multicollinearity of regression models was assessed through a computation of correlation coefficients of independent variables by examining tolerance value or variance inflation factor (VIF). In addition, a likelihood ratio test was used to evaluate the fitness of the final model.

Statistical analysis was conducted with Stata version 14.1 (Statcorp, College Station, TX, USA).

## 3 Results

Of the 2644 participants who were enrolled in the KP‐led Test and Treat study, 2613 were included in the analysis. Thirty‐one participants were excluded from the analysis because they did not complete the risk assessment survey. Of the 2613 participants, 1288 (49.29%) self‐identified as having no or mild risk of HIV infection, and therefore were categorized as having low self‐perceived HIV risk; 1316 (50.36%) participants self‐identified as having moderate or high risk, and were categorized as having high self‐perceived HIV risk.

This paper focuses on the 1288 participants who were categorized as having low self‐perceived HIV risk. In this section, we describe demographic and behavioural characteristics of the 882 MSM and 406 TGW participants stratified by the congruence of their self‐perceived and actual risks, as well as factors that are significantly associated with those whose self‐perceived and actual risk are incongruent.

Of the 882 MSM who were categorized as having low self‐perceived risk of getting HIV, 718 (81.4%) reported to have at least one of our predetermined, actual HIV‐risk characteristics: 15.9% tested HIV positive, 87.1% engaged in condomless sex, 37% tested positive for STIs, and 6.8% had used ATS. This group were+++ classified as risk discordant (RD), while their counterparts – who were categorized as having low self‐perceived risk of acquiring HIV and did not report any predetermined, actual HIV‐risk characteristics – were classified as risk concordant (RC). RD and RC MSM had similar age (median age (IQR): 23.3 (20.5 to 28.5) for RD versus median age (IQR) 24.6 (20.8 to 29.3) for RC) (*p* = 0.09), and the majority of MSM were between 18 and 25 years for both groups (61.6% for RD vs. 53.7% for RC; *p* = 0.06). RD and RC MSM also had similar religion (*p* = 0.28), occupation (*p* = 0.23), and income (*p* = 0.45). More MSM who were RD were located in Chiang Mai (43.3% vs. 22.6%, *p* < 0.001), living with a male partner (25.9% vs. 15.2%, *p* = 0.013), and had less than a bachelor's degree (70.9% vs. 61.7%, *p* = 0.023) when compared to their RC counterparts (Table 1).

**Table 1:** Demographic and behavioural characteristics of MSM stratified by the congruence of self‐perceived versus actual risks (n = 882)

Characteristics	Risk discordant (n = 718) N (%)	Risk concordant (n = 164) N (%)	*p*‐value
Age (years)			
Median (IQR)	23.3 (20.5 to 28.5)	24.6 (20.8 to 29.3)	0.09
Age group			0.06
18 to 25 years old	442 (61.6)	88 (53.7)	
>25 years old	276 (38.4)	76 (46.3)	
Site			
Bangkok	311 (43.3)	69 (42.1)	<0.001
Chiang Mai	260 (36.2)	37 (22.6)	
Hat Yai	71 (9.9)	32 (19.5)	
Pattaya	76 (10.6)	26 (15.9)	
Marital status	n = 714	n = 164	0.013
Single	488 (68.3)	130 (79.3)	
Living with a male partner	185 (25.9)	25 (15.2)	
Ever married with a woman	41 (5.7)	9 (5.5)	
Religion	n = 715	n = 164	0.28
Buddhism	663 (92.7)	148 (90.2)	
Others	52 (7.3)	16 (9.8)	
Education	n = 711	n = 162	0.023
Less than bachelor's degree	504 (70.9)	100 (61.7)	
Bachelor's degree or higher	207 (29.1)	62 (38.3)	
Main occupation	n = 713	n = 163	0.23
Unemployed/student	284 (39.8)	76 (46.6)	
Employed	302 (42.4)	58 (35.6)	
Sex work	127 (17.8)	29 (17.8)	
Monthly income (Thai Baht)	n = 604	n = 136	
Median (IQR)	10,000 (8750 to 15,000)	12,000 (8000 to 18,200)	0.45
Monthly income group			
≤10,000 Thai Baht	332 (55)	64 (47.1)	0.10
>10,000 Thai Baht	272 (45)	72 (52.9)	
Have ever had HIV testing before enrolment			
No	365 (52.1)	67 (41.6)	0.016
Yes	335 (47.9)	94 (58.4)	
Have ever used PrEP			
No/Never known PrEP	704 (98.2)	154 (95.1)	0.039
Yes	13 (1.8)	8 (4.9)	
Have ever used PEP			
No/Never known PEP	698 (97.2)	155 (94.5)	0.08
Yes	20 (2.8)	9 (5.5)	
Age at first sexual intercourse (years)			
Median (IQR)	17 (15 to 19)	18 (16 to 20)	0.038
Age at first sexual intercourse group			
<17 years old	280 (40.1)	55 (34.8)	0.22
≥17 years old	419 (59.9)	103 (65.2)	
Number of sexual partners in the previous six months			
No sexual partner	11 (1.6)	16 (10)	<0.001
Single partner	184 (26.2)	36 (22.5)	
Multiple partners	383 (54.6)	72 (45)	
Had sex in the past six months but did not specify the number of partners	123 (17.5)	36 (22.5)	
Male circumcision			
No	530 (86.2)	125 (83.9)	0.47
Yes	85 (13.8)	24 (16.1)	
Drug used in the past six months			
No	452 (65)	114 (72.6)	0.07
Yes	243 (35)	43 (27.4)	
Had group sex in the past six months			
No	635 (92.4)	152 (96.2)	0.09
Yes	52 (7.6)	6 (3.8)	
Knowledge about getting HIV infection: How to get HIV infection? (Number of correct answers)			
Vaginal or anal sexual intercourse (True)	668 (94.5)	155 (94.5)	
Have meals with an HIV‐infected person (False)	649 (91.8)	157 (95.7)	
Receive blood or blood product that has HIV (True)	415 (58.7)	83 (50.6)	
Share needles with an HIV‐infected person (True)	533 (75.4)	118 (72)	
Kissing on the lips (False)	582 (82.3)	139 (84.8)	
Share toilets with an HIV‐infected person (False)	678 (95.9)	157 (95.7)	
Taking care of an HIV‐infected person (False)	673 (95.2)	159 (97)	
Bitten by mosquitoes (False)	644 (91.1)	151 (92.1)	
Transmit HIV from pregnant mother to infant (True)	370 (52.3)	80 (48.8)	
Score of knowledge about getting HIV infection (Total 9 points)			
Median (IQR)	8 (6 to 8)	7 (6 to 9)	0.56
Score group			
<8 points	337 (47.7)	86 (52.4)	0.27
8 to 9 points	370 (52.3)	78 (47.6)	
Knowledge about protection of HIV: What are the ways that you can decrease your risk of getting HIV infection? (Number of correct answers)			
No sexual intercourse (True)	256 (36.3)	62 (37.8)	
External ejaculation (False)	573 (81.2)	136 (82.9)	
Proper use of condoms with every sexual intercourse (True)	643 (91.1)	151 (92.1)	
Only choose to have sexual intercourse with people who look healthy (False)	615 (87.1)	146 (89)	
Clean vaginal and external genitalia after every sexual intercourse (False)	567 (80.3)	136 (82.9)	
Male circumcision or have sexual intercourse with circumcised men (True)	32 (4.5)	5 (3)	
Score of knowledge about protection from HIV (Total 6 points)			
Median (IQR)	4 (3 to 4)	4 (4 to 4)	0.46
Score group			0.09
<4 points	197 (27.9)	35 (21.3)	
4 to 6 points	509 (72.1)	129 (78.7)	
Attitude about people living with HIV (can select more than one choice)			
Do not like being near that person because of fear of getting HIV (Negative)	42 (5.9)	12 (7.3)	
Rather disgusted because the person likely has HIV‐susceptible behaviour (Negative)	15 (2.1)	2 (1.2)	
Do not feel anything, can live in the same society (Neutral)	522 (73.8)	113 (68.9)	
Pity (Positive)	351 (49.6)	74 (45.1)	
Desire to help (Positive)	250 (35.4)	49 (29.9)	
Summary of attitude about person living with HIV			
Negative attitude	0 (0)	0 (0)	0.11
Positive attitude	126 (17.8)	19 (11.6)	
Neutral attitude	243 (34.4)	66 (40.2)	
Mixed attitude	338 (47.8)	79 (48.2)	

When compared to RC MSM, lower proportions of RD MSM had ever tested for HIV (47.9% for RD vs. 58.4% for RC, *p* = 0.016) or had ever used PrEP prior to enrolling in the study (1.8% for RD vs. 4.9% for RC, *p* = 0.039). Similar proportions had ever used PEP (2.8% for RD vs. 5.5% for RC, *p* = 0.08). RD MSM had lower age at first sex (17 years vs. 18 years, *p* = 0.038) and were more likely to report having multiple sex partners (54.6% vs. 45%, *p* < 0.001). Participation in drug use (35% vs. 27.4%, *p* = 0.07), and group sex (7.6% vs. 3.8%, *p* = 0.09) in the past six months were statistically not different when compared to RC MSM. RD and RC MSM also had similar knowledge score about HIV infection (*p* = 0.56) and HIV prevention (*p* = 0.46), and a large percentage of MSM in both groups had mixed attitudes toward people living with HIV (47.8% for RD vs. 48.2% for RC; *p* = 0.11) (Table 1).

Of 406 TGW who were categorized as having low self‐perceived risk of HIV infection, 332 (81.8%) were classified as RD, of whom 7.2% tested positive for HIV, 90.3% engaged in condomless sex, 34.3% tested positive for an STI, and 6% had used ATS. Similar to MSM, RD TGW were younger (median age (IQR): 23.1 (20.6 to 26.7) vs. 24.1 (21.1 to 28.1), *p* = 0.08) when compared to RC TGW; however, both groups were not statistically different when looking at the following characteristics: religion (*p* = 0.74), education (*p* = 0.91), occupation (*p* = 0.18) and income (*p* = 0.50). However, RD TGW were more likely to be located in Chiang Mai (45.8% vs. 29.7%, *p* = 0.038) and live with a male partner (21.6% vs. 6.8%, *p* = 0.005) when compared to RC TGW (Table 2).

**Table 2:** Demographic characteristics of TGW stratified by the congruence of self‐perceived versus actual risks (n = 406)

Characteristics	Risk discordant n = 332 N (%)	Risk concordant n = 74 N (%)	*p*‐value
Age (years)			
Median (IQR)	23.1 (20.6 to 26.7)	24.1 (21.1 to 28.1)	0.08
Age group			
18 to 25 years old	213 (64.2)	43 (58.1)	0.33
>25 years old	119 (35.8)	31 (41.9)	
Site			
Bangkok	37 (11.1)	7 (9.5)	0.038
Chiang Mai	152 (45.8)	22 (29.7)	
Hat Yai	17 (5.1)	4 (5.4)	
Pattaya	126 (38)	41 (55.4)	
Marital status			
Single	255 (77.5)	69 (93.2)	0.005
Living with a male partner	71 (21.6)	5 (6.8)	
Ever married to a woman	3 (0.9)	0 (0)	
Religion			
Buddhism	320 (96.4)	71 (95.9)	0.74
Others	12 (3.6)	3 (4.1)	
Education			
Less than bachelor's degree	279 (84.3)	62 (83.8)	0.91
Bachelor's degree or higher	52 (15.7)	12 (16.2)	
Main occupation			
Unemployed/student	121 (36.4)	28 (38.4)	0.18
Employed	121 (36.4)	19 (26)	
Sex work	90 (27.1)	26 (35.6)	
Monthly income (baht)			
Median (IQR)	10,000 (8000 to 15,000)	10,000 (10,000 to 15,000)	0.50
Monthly income group			
≤10,000 baht	168 (57.7)	38 (61.3)	0.61
>10,000 baht	123 (42.3)	24 (38.7)	
Have ever had HIV testing before enrolment			
No	146 (45.8)	32 (45.7)	>0.99
Yes	173 (54.2)	38 (54.3)	
Have ever used PrEP			
No/Never known PrEP	326 (98.8)	72 (97.3)	0.30
Yes	4 (1.2)	2 (2.7)	
Have ever used PEP			
No/Never known PEP	326 (98.2)	73 (98.6)	>0.99
Yes	6 (1.8)	1 (1.4)	
Age at first sexual intercourse (years)			
Median (IQR)	16 (15 to 8)	17 (16 to 19)	0.006
Age at first sexual intercourse group			0.005
<17 years old	163 (51.7)	22 (32.8)	
≥17 years old	152 (48.3)	45 (67.2)	
Number of sexual partners in the past 6 months			
No sexual partner	4 (1.2)	12 (16.2)	<0.001
Single partner	80 (24.5)	18 (24.3)	
Multiple partners	144 (44)	24 (32.4)	
Had sex in the past six months but did not specify the number of partners	99 (30.3)	20 (27)	
Male circumcision			
No	236 (91.1)	44 (80)	0.016
Yes	23 (8.9)	11 (20)	
Drug used in the past six months			
No	206 (65.2)	43 (62.3)	0.65
Yes	110 (34.8)	26 (37.7)	
Had group sex in the past six months			
No	296 (92.2)	63 (90)	0.54
Yes	25 (7.8)	7 (10)	
Knowledge about getting HIV infection: How to get HIV infection? (Number of correct answers)			
Vaginal or anal sexual intercourse (True)	306 (92.4)	68 (91.9)	
Have meals with an HIV‐infected person (False)	314 (94.9)	74 (100)	
Receive blood or blood product that has HIV (True)	164 (49.5)	39 (52.7)	
Share needles with an HIV‐infected person (True)	229 (69.2)	61 (82.4)	
Kissing on the lips (False)	288 (87)	68 (91.9)	
Share toilets with an HIV‐infected person (False)	324 (97.9)	74 (100)	
Taking care of an HIV‐infected person (False)	322 (97.3)	73 (98.6)	
Bitten by mosquitoes (False)	304 (91.8)	71 (95.9)	
Transmit HIV from pregnant mother to infant (True)	127 (38.4)	37 (50)	
Score of knowledge about getting HIV infection (Total 9 points)			
Median (IQR)	7 (6 to 8)	8 (7 to 9)	0.002
Score group			
<8 points	194 (58.6)	26 (35.1)	<0.001
8 to 9 points	137 (41.4)	48 (64.9)	
Knowledge about protection from HIV: What are the ways that you can decrease your risk of getting HIV infection? (Number of correct answers)			
No sexual intercourse (True)	98 (29.7)	19 (26)	
External ejaculation (False)	277 (83.9)	60 (82.2)	
Proper use of condoms with every sexual intercourse (True)	300 (90.9)	71 (97.3)	
Only choose to have sexual intercourse with people who look healthy (False)	307 (93)	69 (94.5)	
Clean vaginal and external genitalia after every sexual intercourse (False)	276 (83.6)	59 (80.8)	
Male circumcision or have sexual intercourse with circumcised men (True)	8 (2.4)	2 (2.7)	
Score of knowledge about protection from HIV (Total 6 points)			
Median (IQR)	4 (4 to 4)	4 (3 to 4)	0.88
Score group			
<4 points	81 (24.5)	19 (26)	0.79
4 to 6 points	249 (75.5)	54 (74)	
Attitude about person living with HIV infection (can select more than 1 choice)			
Do not like being near that person because of fear of getting HIV (Negative)	34 (10.3)	5 (6.8)	
Rather disgusted because the person likely has HIV‐susceptible behaviour (Negative)	5 (1.5)	2 (2.7)	
Do not feel anything, can live in the same society (Neutral)	247 (74.8)	53 (72.6)	
Pity (Positive)	171 (51.8)	41 (56.2)	
Desire to help (Positive)	131 (39.7)	32 (43.8)	
Summary of attitude about person living with HIV			
Negative attitude	0 (0)	0 (0)	0.53
Positive attitude	81 (24.5)	16 (21.9)	
Neutral attitude	110 (33.3)	21 (28.8)	
Mixed attitude	139 (42.1)	36 (49.3)	

There was no difference in HIV testing history and PrEP or PEP use between RD and RC TGW. Nearly 46% of RD TGW and 45.7% of RC TGW were first‐time HIV testers (*p *> 0.99). Similar to their MSM counterparts, the majority of TGW in both groups never used PrEP (98.8% for RD vs. 97.3% for RC; *p* = 0.30) or PEP (98.2% for RD vs. 98.6% for RC; *p*> 0.99). Age of sexual debut was lower among RD TGW (median (IQR): 16 (15 to 18)) compared to RC TGW (median (IQR): 17 (16 to 19); *p* = 0.006). Similar proportions of RD and RC TGW reported drug use (*p* = 0.65) and group sex (*p* = 0.54) in the past six months. A higher proportion of RD TGW (51.7%) had their first sexual encounter when they were less than 17 years old, compared to 32.8% of RC TGW (*p* = 0.005). In the past six months, a higher proportion of RD TGW reported having multiple sex partners (44% vs. 32.4%, *p* < 0.001). RD TGW had a lower median score of knowledge about HIV transmission compared to RC TGW (*p* = 0.002), but there was no difference in knowledge about HIV prevention (*p* = 0.88). Similar to MSM, almost half of TGW in both groups had mixed attitudes toward people living with HIV (*p* = 0.53) (Table 2).

Logistic regression found that living with a male partner (aOR: 2.0; 95% CI: 1.2 to 3.2; *p* = 0.005), having never tested for HIV (aOR: 1.5; 95% CI: 1.0 to 2.1; *p* = 0.045), having a positive attitude toward people living with HIV (aOR: 1.9; 95% CI: 1.9 to 3.4; *p* = 0.032), and living in Bangkok (aOR: 2; 95% CI: 1.2 to 3,4; *p* = 0.01) and Chiang Mai (aOR: 2.8; 95% CI: 1.6 to 4.9; *p* < 0.001) were associated with increased risk discordance between self‐perceived risk and actual risk among MSM participants (Table 3).

**Table 3:** Factors associated with risk discordance among MSM

Factors	Univariate			Multivariable		
	OR	95% CI	*p*‐value	aOR	95% CI	*p*‐value
Age at enrolment						
18 to 25 years old	1.4	1 to 1.9	0.06	1.2	0.8 to 1.9	0.39
>25 years old	Ref.			Ref.		
Site						
Bangkok	2	1.2 to 3.3	0.005	2	1.2 to 3.4	0.01
Chiang Mai	3.2	1.8 to 5.4	<0.001	2.8	1.6 to 4.9	<0.001
Hat Yai	Ref.			Ref.		
Pattaya	1.3	0.7 to 2.4	0.38	1.1	0.5 to 2.2	0.85
Marital status						
Single	Ref.			Ref.		
Living with a male partner	2	1.2 to 3.1	0.004	2	1.2 to 3.2	0.005
Married to a woman and living together/divorced or separated	1.2	0.6 to 2.6	0.61	1	0.5 to 2.3	0.94
Education						
Less than Bachelor's degree	1.5	1.1 to 2.2	0.023	1.2	0.8 to 1.9	0.32
Bachelor's degree or higher	Ref.			Ref.		
Main occupation						
Employed	Ref.			Ref.		
Unemployed/student	0.7	0.5 to 1	0.09	0.9	0.5 to 1.4	0.64
Service worker	0.8	0.5 to 1.4	0.49	1.1	0.6 to 2.1	0.71
Monthly income						
≤10,000 baht	1.4	0.9 to 2	0.10			
>10,000 baht	Ref.					
Have ever had HIV testing before enrolment						
No	1.5	1.1 to 2.2	0.016	1.5	1 to 2.1	0.045
Yes	Ref.			Ref.		
Age at first sexual intercourse						
<17 years old	1.3	0.9 to 1.8	0.22			
≥17 years old	Ref.					
Male circumcision						
No	1.2	0.7 to 2	0.47			
Yes	Ref.					
Score of knowledge about getting HIV infection (8 points)						
<8 points	0.8	0.6 to 1.2	0.27			
≥8 points	Ref.					
Score of knowledge about protection from HIV (4 points)						
<4 points	1.4	0.9 to 2.1	0.09	1.2	0.8 to 1.9	0.38
≥4 points	Ref.			Ref.		
Attitude about people living with HIV						
Neutral attitude	Ref.			Ref.		
Positive attitude	1.8	1 to 3.1	0.037	1.9	1.1 to 3.4	0.032
Mixed attitude	1.2	0.8 to 1.7	0.42	1.2	0.8 to 1.7	0.39

Our logistic regression model found that the following factors increase risk discordance among TGW participants: living with a male partner (aOR: 5.6; 95% CI: 1.9 to 16.4; *p* = 0.002), age of sexual debut less than 17 years old (aOR: 2.7; 95% CI: 1.5 to 4.9; *p* = 0.001), and scoring less than eight points in HIV transmission knowledge (aOR: 2.9; 95% CI: 1.6 to 5.1; *p* < 0.001) (Table 4). Being a sex worker (aOR: 0.5; 95% CI: 0.2 to 0.9; *p* = 0.034) was found to decrease the chance of risk discordance among TGW (Table 4).

**Table 4:** Factors associated with risk discordance among TGW

Factors	Univariate			Multivariable		
	OR	95% CI	*p*‐value	aOR	95% CI	*p*‐value
Age at enrolment						
18 to 25 years old	1.3	0.8 to 2.2	0.33			
>25 years old	Ref.					
Site						
Bangkok	1.2	0.3 to 4.8	0.75			
Chiang Mai	1.6	0.5 to 5.3	0.42			
Hat Yai	Ref.					
Pattaya	0.7	0.2 to 2.3	0.58			
Marital status						
Single	Ref.			Ref.		
Living with a male partner	3.8	1.5 to 9.9	0.005	5.6	1.9 to 16.4	0.002
Married to a woman and living together/divorced or separated	(empty)			(empty)		
Education						
Less than Bachelor's degree	1	0.5 to 2.1	0.91			
Bachelor's degree or higher	Ref.					
Main occupation						
Employed	Ref.			Ref.		
Unemployed/student	0.7	0.4 to 1.3	0.23	0.5	0.3 to 1.1	0.07
Service worker	0.5	0.3 to 1	0.07	0.5	0.2 to 0.9	0.034
Monthly income						
≤10,000 baht	0.9	0.5 to 1.5	0.61			
>10,000 baht	Ref.					
Have ever had HIV testing before enrolment						
No	1	0.6 to 1.7	>0.99			
Yes	Ref.					
Age at first sexual intercourse						
<17 years old	2.2	1.3 to 3.8	0.006	2.7	1.5 to 4.9	0.001
≥17 years old	Ref.			Ref.		
Male circumcision						
No	2.6	1.2 to 5.6	0.019			
Yes	Ref.					
Score of knowledge about getting HIV infection (9 points)						
<8 points	2.6	1.5 to 4.4	<0.001	2.9	1.6 to 5.1	<0.001
≥8 points	Ref.			Ref.		
Score of knowledge about protection from HIV (6 points)						
<4 points	0.9	0.5 to 1.7	0.79			
≥4 points	Ref.					
Attitude about person with HIV infection						
Neutral attitude	Ref.					
Positive attitude	1	0.5 to 2	0.93			
Mixed attitude	0.7	0.4 to 1.3	0.31			

## 4 Discussion

Self‐perceived HIV risk is an important precursor of health‐seeking behaviour. The discordance between self‐perceived and actual risk may put RD KPs at heightened HIV risk. Among MSM who were RD, 15.9% tested HIV‐positive, 87.1% engaged in condomless sex in the past six months, 37% tested positive for an STI, and 6.8% had used ATS (injection or non‐injection). When compared to other studies, our numbers are higher for those who participated in condomless sex (18% to 76%) 9, 19, 20, 21, 22 but similar for STI diagnosis (21% to 39%) 9, 19, 20, 22. Moreover, a considerable number of MSM who perceived themselves to be at low risk for acquiring HIV were found to be HIV positive, but unaware of their status 9, 20. Risk discordance among TGW has not been reported previously in the literature, but our data shows high proportions of RD TGW who engaged in HIV‐susceptible behaviours.

We also found that living with a male partner was correlated with risk discordance for both MSM and TGW, which could result from a monogamous relationship in which the participants knew their partner's HIV status or the inconsistent condom use when these individuals engage in a trusted, intimate, stable relationship 23, 24. Past literature reported that MSM in exclusively male partnerships and MSM with a mix of casual, regular and commercial partners had lower consistency in condom use than MSM with partners of both genders 25, with the lowest consistency in condom use reported among MSM and their regular partner 23, 26, 27. Condom use with regular partners has been observed to be a challenge, despite it being the main HIV protection method in Thailand 28. However, these participants might choose to use alternative protection methods, such as serosorting, taking PrEP, and/or having sex with partner with undetectable viral load, which were not accounted for in this analysis. Hence, this might inflate the RD group. Nonetheless, these alternative protection methods were not widespread and well known during the time of the study (2015 to 2016). 28, 29, 30, 31

For MSM, having never tested for HIV and having a positive attitude toward PLHIV were associated with RD. This could be that, in the era of ART, life expectancy of people living with HIV is not severely shortened, and it is seen as a chronic condition, instead of a terminal illness 32. Thus, people may be more susceptible to partaking in HIV‐susceptible behaviours. Past studies showed men who were more optimistic about HIV treatment engaged in more HIV‐risk behaviours and vice versa 32, 33, 34. Additionally, low self‐perceived risk may be the underlying reason why these individuals never sought HIV testing before they enrolled in this study 35, 36, 37. A proactive approach among health care workers to HIV testing is needed to encourage people at heightened risk to get tested 36, 38.

TGW sex workers were less likely to underestimate their risk of acquiring HIV, which could be due to the apparent nature of the work. However, having sexual debut at less than 17 years and a low knowledge score in HIV transmission were positively associated with the incongruence of dfperceived and actual risk. Previous Thai study found that earlier sexual debut was associated with prevalent HIV infection 39, which could be a result of limited knowledge about HIV transmission, leading to engaging in condomless sex 40, 41. Despite our findings, it is crucial to tailor educational materials to TGW preferences; they are more likely to allow fellow TGW peers who they can identify with to educate them 42.

Interestingly, MSM and TGW in RD and RC groups shared similar characteristics. Accurate risk perception was not affected by age, religion, occupation, and income in both MSM and TGW. PEP use, drug use, and group sex experience did not affect risk perception in MSM, while HIV testing, PrEP or PEP use, drug use, and group sex experience did not affect risk perception of TGW. Although not statistically significant in our analysis, a study conducted in Vietnam suggested that these behaviours are indicative of their sexual patterns that make them susceptible to HIV infection 43. Individuals presented with any of these characteristics should be advised and offered HIV prevention methods. For example, those who take PEP were exposed to HIV risk, so healthcare providers may suggest HIV prevention methods, such as PrEP or male circumcision 44. However, this makes it challenging to distinguish individuals who actually have high likelihood of HIV infection apart from all those who perceived themselves as having low risk. While the knowledge of HIV/AIDS may facilitate accurate self‐risk assessment, our finding supported previous systematic review, which did not find this association 45. This could be that people cannot perceive the risk when they have not much knowledge, and an increase in knowledge to a certain point may improve risk perception; however, further increase in knowledge may reduce risk perception, because knowledge may produce biases in judgment, such as optimistic bias, psychological distancing and overconfidence, leading to inaccurate risk perception 45.

Our study had several limitations. Our findings are based on MSM and TGW populations who are in urban settings, and it is not clear how applicable our results may be. Moreover responses may have been subjected to recall bias, and risks and risk assessment may change over time. We also assumed that individuals having participated in at least one HIV‐susceptible behaviour were in risk discordant group, which might exaggerate the number of participants in the risk discordant group. Moreover while there are past studies on this topic among MSM, TGW are understudied, making it challenging to validate our findings. Therefore, more related studies on TGW are needed. Furthermore, our study did not directly measure stigma associated with gender identity, sexual orientation, or HIV, and thereby could not analyse how these may mediate risk‐perception among our participants.

## 5 Conclusions

Over 80% of our MSM and TGW participants perceived themselves as having low HIV risk even though they actually undertook considerable HIV‐related risks. Clarifying self‐perceived risk versus actual risk may make HIV‐vulnerable populations more proactive and positively enhance their health‐seeking behaviours. It is important to evaluate at the macro‐level how structural factors create a barrier that deter those at heightened risk from accessing necessary health‐promoting resources. Developing targeted individual‐level interventions without contextualizing risk factors, for example, analysing why they are at risk in the first place, can only exacerbate the degree of health inequalities in marginalized populations.

## Competing interests

All authors declare no competing interests related to this work.

## Authors' contributions

PS drafted the manuscript and developed the analysis plan. SP, JJ and NP led the study. TS, WS, ST, PP, SS, PP, SK and PP conducted the study in their respective sites. PS, SL, RM, and PM analysed the data. SM, MA, SC and RV approved the study. All authors provided critical reviews of the manuscript. PS and SL revised the manuscript based on the comments received. PP and NP approved the final version of the manuscript.
